# A Scoping Review of the Use and Determinants of Social Media Among College Students

**DOI:** 10.3390/healthcare13172234

**Published:** 2025-09-06

**Authors:** Anam Fatima, Md. Sohail Akhter, Amar Kanekar, Sharmistha Roy, Rupam Mitra, Blessing Imade, Manoj Sharma

**Affiliations:** 1 Department of Social and Behavioral Health, School of Public Health, University of Nevada, Las Vegas, NV 89119, USA; fatima1@unlv.nevada.edu (A.F.); akhtem1@unlv.nevada.edu (M.S.A.); roys5@unlv.nevada.edu (S.R.); mitrar1@unlv.nevada.edu (R.M.); imade@unlv.nevada.edu (B.I.); manoj.sharma@unlv.edu (M.S.); 2 School of Counseling, Human Performance and Rehabilitation, University of Arkansas at Little Rock, Little Rock, AR 72204, USA; 3 Department of Internal Medicine, Kirk Kerkorian School of Medicine at UNLV, University of Nevada, Las Vegas, NV 89102, USA

**Keywords:** social media, students, mental health, behavior, addictive, anxiety

## Abstract

**Background/Objectives**: Use of social media among college students is ubiquitous. Excessive use of social media has been linked to distractions, reduced academic focus, and poor mental health outcomes such as anxiety and depression. The determinants of social media use among college students are not well understood. Hence, the purpose of this study was to conduct a scoping review on the behavioral, demographic, and psychosocial determinants, explore theoretical frameworks, and suggest evidence-based recommendations. **Methods**: This scoping review was conducted between January 2024 and May 2025 following PRISMA-ScR guidelines, using MEDLINE (PubMed), CINAHL, and ERIC databases. Peer-reviewed studies were included if they focused on college students (ages 18–30), investigated determinants of social media use, and met predefined inclusion criteria. **Results**: A total of 22 studies met the inclusion criteria. Studies were conducted in Bangladesh, Canada, China, Egypt, India, Nigeria, Pakistan, Saudi Arabia, Turkey, and the United States, and the majority used cross-sectional designs (n = 20). A consistent finding across the reviewed studies was the strong association between social media overuse and symptoms of depression, anxiety, stress, and emotional dysregulation. Very few theoretical frameworks for understanding the determinants of social media were used. According to the reviewed studies, factors such as fear of missing out, sleep quality, and prolonged social media use consistently emerged as significant predictors of adverse mental health outcomes (*p* < 0.05). **Conclusions**: In this study, problematic social media use (PSMU) was linked to increased mental health issues, suggesting that students frequently engage in social comparison and experience feelings of missing out (FoMO), which exacerbate emotional distress. There is a need for integrated approaches in addressing PSMU within educational environments, particularly in fostering healthier digital habits among students. There is a need to conduct more concerted research using longitudinal designs and contemporary theoretical frameworks in this area.

## 1. Introduction

Social media is an integral part of modern life, especially for college students. These platforms shape communication, relationships, and engagement with information and health. In the U.S., about 93% of individuals aged 18–24 use at least one social media platform [1]. Social media platforms such as Facebook, Instagram, Twitter, WhatsApp, LinkedIn, and YouTube offer multimodal interaction through text, visuals, and videos [2,3]. They influence academic collaboration, self-expression, and relationship maintenance [4,5].

Social media platforms have their roots in the early 2000s, beginning with sites like Friendster and MySpace, which paved the way for the rise of global networks such as Facebook and Instagram [6,7]. These platforms transformed online communication by enabling users to share dynamic content, explore identities, and connect with peers in more interactive ways [8,9]. Beyond personal socialization, social media now plays an important role in civic engagement, emotional support, professional networking, and even mental health assistance [10,11,12,13]. This expanding role highlights the diverse impact of social media on college students’ lives and sets the stage to explore its effects in greater detail.

For this review, ‘social media’ refers to web- and mobile-based platforms that facilitate user-generated content, interaction, and sharing (e.g., Facebook, Instagram, TikTok) [6]. ‘Usage’ includes both passive consumption and active engagement behaviors. ‘Addiction’ is understood as excessive or compulsive use characterized by impaired control and negative consequences [10,11]. Determinants refer to demographic, behavioral, psychological, and contextual factors associated with social media use.

This review distinguishes between social media use and broader mobile or smartphone use. While some included studies examined general smartphone behavior, our synthesis focuses on social media platforms that enable interaction and content-sharing, such as Instagram, Twitter, Facebook, and TikTok.

College students spend an average of two to four hours daily on social media [14]. Used constructively, it facilitates peer interaction and enhances academic performance [5]. However, excessive use has been linked to distractions, reduced academic focus, and poor mental health outcomes such as anxiety and depression [15,16,17]. Problematic usage may result in addictive behaviors, privacy concerns, and cyberbullying [18,19,20].

Measuring social media use remains complex, as many studies rely on self-reported duration or frequency, which may be biased [21]. Tools like the Bergen Social Media Addiction Scale (BSMAS) assess addiction symptoms, while the Social Media Use Scale (SMUS) classifies usage into image-, comparison-, belief-, and consumption-based domains [22]. These tools are useful for understanding patterns and predictors of behavior. Social media also affects sleep quality, emotional regulation, and screen time habits [19,20]. During the COVID-19 pandemic, students experienced heightened social media use, which altered engagement behaviors and increased susceptibility to problematic patterns [13]. Social media screening tools like HEADS4 have also been used in adolescent primary care to identify potential behavioral health issues [17].

To promote healthier digital habits, scholars advocate for media literacy education, wellness campaigns, and screen-time monitoring apps [8,23]. Online peer communities and student-led support groups may reduce feelings of isolation and foster belongingness [5,11]. Nonetheless, gaps in theoretical application persist. While some studies explore constructs such as self-esteem, peer influence, and academic pressure [7,24], only a few employ structured behavioral theories or comprehensive models to explain use. Despite extensive research, literature remains fragmented and often lacks theoretical frameworks. Many studies are descriptive and do not apply behavior change theories such as the Theory of Planned Behavior (TPB), the Health Belief Model (HBM), or the Multi-Theory Model (MTM) to explain social media use. A scoping review can identify these evidence gaps and support theory-driven interventions [25,26].

Unlike the 2023 systematic review by Keles et al. [25], which focused primarily on outcomes such as depression and anxiety associated with social media use, this scoping review emphasizes the determinants of social media behavior among college students. It also highlights the limited application of behavioral theories in current literature and includes only post-2023 studies (2024–2025) to capture the most recent trends in digital engagement. This focused lens allows for a more theory-informed, determinant-driven synthesis relevant to designing effective interventions.

This scoping review aims to synthesize literature on social media use among college students. It identifies behavioral, demographic, and psychosocial determinants, explores theoretical frameworks, and suggests evidence-based recommendations to support healthy digital engagement.

## 2. Materials and Methods

### 2.1. Protocol

The scoping review was performed following the guidelines outlined in the Preferred Reporting Items for Systematic Reviews and Meta-Analyses—Extension for Scoping Reviews [27] (PRISMA-ScR) statement. The search was conducted between January 2024 and May 2025. This review was not registered.

### 2.2. Eligibility Criteria

The eligibility criteria for this scoping review were established following the PRISMA-ScR guidelines. The review focused on studies published in peer-reviewed journals within the selected databases, including MEDLINE (PubMed), CINAHL, and ERIC. The inclusion criteria were as follows: (a) the target population comprised college students, (b) only studies published in peer-reviewed journals were considered, (c) studies investigating determinants, antecedents, correlates, or factors influencing social media use, (d) primary studies with both qualitative and quantitative research designs, and (e) articles published in English. The exclusion criteria included: (a) gray literature, (b) studies not indexed in the selected databases, (c) studies involving non-college-going youth, and (d) review articles. Additionally, to ensure the relevance and currency of the evidence, the review included studies published between January 2024 and May 2025, as a systematic review on the topic had already been conducted in 2023 [28]. Included studies involved college students aged 18–30 and examined behavioral, demographic, or psychosocial determinants of social media use. The inclusion/exclusion criteria are summarized in Table 1.

### 2.3. Search Strategy

The search terms for this scoping review were designed to address the key concepts of the research question: “social media use,” “college students,” and “determinants”. These terms were carefully selected and adapted by one author, with verification and discussion among the other authors. Given that literature often does not distinguish between similar terms such as “social media” and “social networking sites,” the search included variations to ensure comprehensive retrieval of relevant studies. The search strings used for PubMed, ERIC, and CINAHL were as follows:

For PubMed, the search incorporated MeSH terms for social media and college students, along with relevant terms for behavior and its determinants: 

(“social media”[MeSH Terms] OR “social media” OR “social networking sites” OR “online social networks”) AND (“college students” OR “university students”) AND (“determinants” OR “factors” OR “influences”) AND (“use” OR “usage” OR “engagement” OR “behavior”)

For ERIC and CINAHL, the search used keyword-based queries for social media and college students, including terms related to behavior and influencing factors:

(“social media” OR “social networking sites” OR “online social networks”) AND (“college students” OR “university students”) AND (“determinants” OR “factors” OR “influences”) AND (“use” OR “usage” OR “engagement” OR “behavior”)

This approach ensured that all relevant articles related to social media use among college students and the factors influencing such behavior were captured across the selected databases.

### 2.4. Selection Process

Two authors independently screened the titles and abstracts for eligibility. Full texts were then reviewed for inclusion. Discrepancies or disagreements were resolved through discussion and consensus among the review team. Excel was used for charting, and the table was collaboratively developed by all authors, following the guidelines of the Preferred Reporting Items for Systematic Reviews and Meta-Analyses Extension for Scoping Reviews (PRISMA-ScR) checklist. The data extracted from each study included the title, publication date, country/region, author(s), study design, target population, sample size, key findings, and limitations in the research. Key findings primarily focused on the factors affecting social media use among college students. The use of green, red, and yellow coding was employed for paper selection.

In our review, data of each included study was charted using an excel sheet. Two reviewers independently extracted the data to minimize bias, and any discrepancies were resolved through discussion or consultation with a third reviewer. Additional details were sought from study authors when necessary, and extracted information was cross verified for accuracy. This process ensured completeness and reliability.

**Figure 1:**
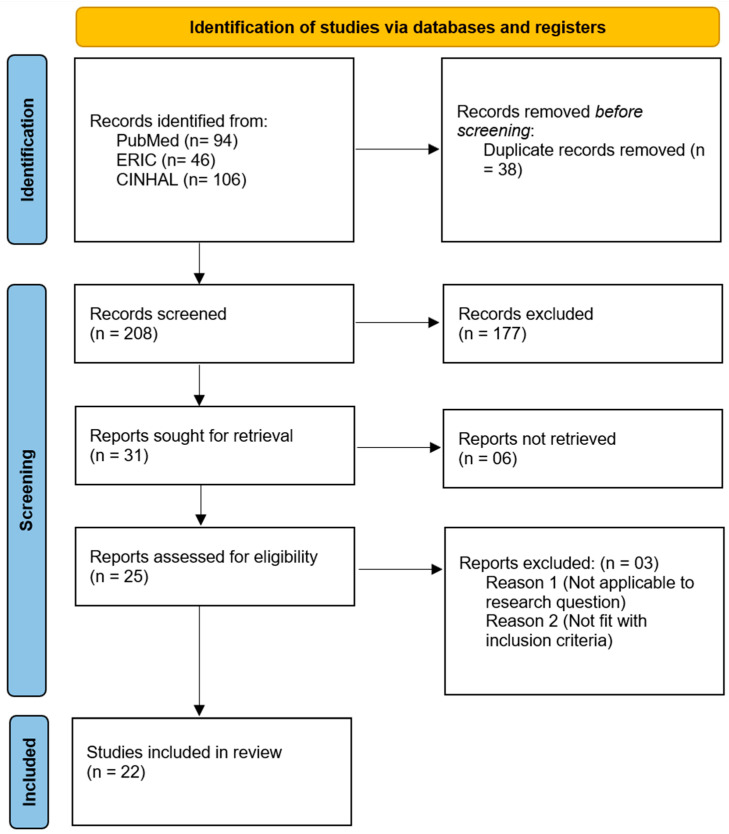
PRISMA flowchart.

We extracted data on study characteristics (author, year, country, study design, sample size), participant demographics (age, gender, education level), and key variables related to social media use (duration, frequency, platforms used, purpose, and patterns). We also charted determinants such as fear of missing out (FoMO), sleep quality, mental health indicators (stress, anxiety, depression), and lifestyle behaviors. For consistency, we assumed self-reported measures reflected actual behavior unless otherwise noted and simplified multi-item scales by recording their overall composite scores (Figure 1).

## 3. Results

A total of 22 studies met the inclusion criteria. It was found that problematic social media use (PSMU), smartphone addiction, fatigue, and associated psychological outcomes among university students were common. The research spanned diverse cultural and geographical contexts, with studies from Bangladesh, Canada, China, Egypt, India, Nigeria, Pakistan, Saudi Arabia, Turkey, and the United States. Most studies employed cross-sectional designs (n = 20), with two longitudinal or comparative surveys. Sample sizes ranged from 389 to 1,136, and participants were primarily undergraduate or graduate students aged between 18 and 30. The characteristics of the included studies are presented in Table 2. Table 3 summarizes the key determinants and main findings from each study. While findings varied, several core themes emerged, including emotional dysregulation, social comparison, loneliness, and academic stress. These determinants interacted across cognitive, behavioral, and contextual levels.

One of the most consistent findings across the reviewed studies was the strong association between social media overuse and symptoms of depression, anxiety, stress, and emotional dysregulation. For instance, Shen et al. (2024) [29] applied a network analysis approach to reveal how anxiety and depression symptoms were directly interlinked with problematic smartphone behaviors. Similarly, Sirtoli et al. (2024) [30] found that depressive symptoms significantly mediated the relationship between excessive social media use and substance abuse among Spanish students. Jameel et al. (2025) [31] and Wang et al. (2024) [32] reported that psychological distress, including depression and anxiety, served both as predictors and outcomes of excessive digital engagement. Fruehwirth et al. (2023) [33] further highlighted that the COVID-19 pandemic intensified psychological vulnerability, and peer support moderated these effects.

Several studies also focused on upward social comparison and fear of missing out (FoMO) as primary predictors of compulsive online behavior. Yan et al. (2024) [34] showed that students experiencing frequent social comparison and cognitive overload were more likely to report depressive mood and stress. Thomas (2024) [35] found FoMO to be one of the strongest psychological motivators of problematic social media use, along with emotional distress. Üzer et al. (2024) [36] contributed a novel perspective by linking psychological pain, a component of suicidality, with chronotype and social media engagement, underscoring the emotional intensity embedded in online experiences.

The internal dimensions of self-concept, particularly low self-esteem, emotional intelligence, and alexithymia, were frequently reported as mediators or predictors of problematic social media use. Eymirli et al. (2024) [37] demonstrated that low self-esteem and loneliness significantly predicted problematic social media behaviors among Turkish university students. Li et al. (2024) [38] and Helmy et al. (2024) [39] emphasized the mediating role of alexithymia, or difficulty in identifying and expressing emotions, suggesting that individuals with impaired emotional awareness may resort to excessive online activity as a coping mechanism. Yuan et al. (2024) [40] found that low emotional intelligence weakened students’ ability to cope with stress, thereby increasing reliance on digital engagement.

Studies consistently identified behavioral metrics, particularly excessive screen time, frequency of platform use, and smartphone dependence, as core determinants. Rahman et al. (2025) [41] found that higher scores in social media addiction (SMA), fatigue (SMF), and FoMO were all correlated with poor sleep quality and excessive use. Bawazeer et al. (2024) [42] introduced the concept of nomophobia as a powerful predictor of addiction, suggesting that fear of being without one’s phone contributes to compulsive behavior.

Several studies also explored contextual and environmental factors. Gao et al. (2024) [43] and Wang et al. (2024) [32] highlighted that poor sleep quality significantly mediated the relationship between social media use and psychological symptoms, with late-night usage being particularly harmful. Pi et al. (2024) [44] examined academic stress and found it to be positively associated with social media fatigue and compulsive behaviors. In addition, Sun et al. (2024) [45], who concentrated on gender dynamics and peer norms, students were more susceptible to addictive behaviors because of the strong influence of peer-driven online norms and social acceptance. Furthermore, Oyinbo et al. (2024) [46] connected problematic smartphone use and academic procrastination to poor cognitive emotion regulation techniques, suggesting that emotional coping deficiencies may cause excessive dependence on digital platforms.

**Table 2:** Descriptive Table of Characteristics of Studies.

Study (Author, Year)	Country	Study Design	Sample Size	Population	Social Media Platform(s)
Jameel et al., 2025 [31]	Pakistan	Cross-sectional	600	University Students	Facebook and WhatsApp
Karaduman et al., 2025 [47]	Turkey	Cross-sectional	658	University Students	General social media
Rahman et al., 2025 [41]	Bangladesh	Cross-sectional	611	University Students	Facebook and Messenger
Sun & Tang, 2025 [45]	China	Cross-sectional	431	University Students	WeChat, QQ, and Douyin
Thomas & George, 2025 [35]	India	Cross-sectional	508	University Students	Instagram and WhatsApp
Bawazeer et al., 2024 [42]	Saudi Arabia	Cross-sectional	389	University Students	Snapchat and Instagram
Eymirli et al., 2024 [37]	Turkey	Cross-sectional	928	University Students	Instagram and Twitter
Fruehwirth et al., 2024 [33]	USA	Cross-sectional	2144	University Students	Instagram, Snapchat, TikTok, and Twitter
Gao et al., 2024 [43]	USA	Longitudinal	193	College students with disabilities	Social networking platforms
Ghaderi, 2024 [48]	Iran	Cross-sectional	1317	University Students	General social media
Helmy et al., 2024 [39]	Egypt, Oman, Pakistan	Cross-national	2616	University Students	Smartphone and social media use
Lerma & Cooper, 2024 [20]	Spain	Cross-sectional	2280	University Students	General social media use
Li et al., 2024 [38]	China	Cross-sectional	2582	University Students	General social media
Oyinbo et al., 2024 [46]	Nigeria	Cross-sectional	1416	University Students	General social media
Pi et al., 2024 [44]	China	Cross-sectional	710	University Students	Sina Weibo and WeChat
Sánchez-Fernández et al., 2024 [49]	Spain	Cross-sectional	690	University students	General social media, gaming platforms
Shen et al., 2024 [29]	China	Longitudinal	5568	University freshmen	Mobile phones (general usage)
Sirtoli et al., 2024 [30]	Brazil	Cross-sectional	3161	University Students	General social media
Üzer et al., 2024 [36]	Turkey	Cross-sectional	571	University Students	General social media
Wang et al., 2024 [32]	China	Cross-sectional	3236	University Students	Smartphones
Yan et al., 2024 [34]	China	Cross-sectional	2507	University students	Smartphones
Yuan et al., 2024 [40]	China	Cross-sectional	1294	University students	Smartphones (general use)

**Table 3:** Summary of Key Determinants.

Study (Author, Year)	Aim of the Study	Key Determinants	Main Findings
Jameel et al., 2025 [31]	To analyze the association between social media addiction (SMA) and mental health issues (e.g., depressive symptoms) among Saudi university students, and explore insomnia’s mediating role.	Time spent, depression, and anxiety	Social media addiction directly predicted mental health issues (β = 0.315, *p* < 0.001) and insomnia (β = 0.537, *p* < 0.001). Insomnia mediated the SMA–mental health link (indirect β = 0.187, *p* < 0.001), explaining 18.7% of the effect.
Karaduman et al., 2025 [47]	To assess social-media-addiction levels among nursing and midwifery undergraduates, identify influencing factors, and test whether addiction predicts mental fatigue	Loneliness and fear of missing out (FoMO)	Social-media addiction scores were generally low but still predicted higher mental fatigue (*p* < 0.01). In regression, the mood-regulation and conflict components of addiction each showed significant contributions to fatigue (*p* = 0.011; *p* < 0.001), together explaining a meaningful share of variance.
Rahman et al., 2025 [41]	To examine the associations between social media addiction (SMA), social media fatigue (SMF), fear of missing out (FoMO), and sleep quality (SQ) among university students in Bangladesh.	SMA, SMF, and FoMO	Students with lower SMA, SMF, and FoMO scores were significantly more likely to have good sleep quality (AORs: 2.04, 6.85, 2.22, respectively; all *p* < 0.001). Those spending over 8 h/day on social media had significantly poorer sleep (AOR = 0.20; *p* < 0.001) and worse self-reported health.
Sun & Tang, 2025 [45]	To revise and validate the Problematic Smartphone Use Scale for Chinese college students (PSUS-C) and test its psychometric properties and measurement invariance across demographic groups.		PSUS-C demonstrated strong criterion validity: positively correlated with depression (r = 0.451, *p* < 0.001), loneliness (r = 0.455–0.504, *p* < 0.001), social media addiction (r = 0.614, *p* < 0.001), and phone usage duration (r = 0.148, *p* < 0.001); negatively correlated with life satisfaction (r = −0.218, *p* < 0.001) and self-esteem (r = −0.416, *p* < 0.001).
Thomas & George, 2025 [35]	To examine relationships between Fear of Missing Out (FoMO), emotional distress, and problematic social media use (PSMU) among university students in India.	FOMO, anxiety, and emotional distress	FoMO significantly correlated with depression (r = 0.320, *p* < 0.05), anxiety (r = 0.326, *p* < 0.05), and stress (r = 0.317, *p* < 0.05). Females reported higher anxiety than males (*p* = 0.007).
Bawazeer et al., 2024 [42]	To examine the relationship between social media use and dietary habits among college students in Riyadh, Saudi Arabia.	Nomophobia (the fear or anxiety associated with being without a mobile phone or unable to use it) and daily usage	Students spending ≥ 4 h/day on social media had significantly poorer dietary habits (*p* = 0.029). Significant differences in dietary scores were noted for students without children (*p* = 0.029), without medical issues (*p* = 0.039), and those following specific dietary plans (*p* < 0.001), suggesting negative impacts from extensive social media usage.
Eymirli et al., 2024 [37]	To evaluate healthy lifestyle behaviors, physical activity levels, and social media use among dental students.	Self-esteem and loneliness	Most students (64.3%) had low physical activity; 23.1% were inactive. Higher lifestyle scores correlated positively with increased physical activity (*p* < 0.001). No gender differences in lifestyle behaviors or physical activity. Males showed significantly higher obesity and tobacco use (*p* < 0.05).
Fruehwirth et al., 2024 [33]	To assess the causal effects of social media use on mental health (depression and anxiety) among college students during the COVID-19 pandemic.	Loneliness, depressive symptoms, and social media use frequency	Social media use had no significant effect on mental health 4 months into the pandemic, but significantly worsened depression (β = 0.54, *p* < 0.05) and anxiety (β = 0.47, *p* < 0.10) symptoms at 18 months. Effects were strongest among socially isolated students (*p* < 0.05).
Gao et al., 2024 [43]	Track one-year changes in social media addiction and its impact on career networking among college students with disabilities.	Addiction levels, networking use, and disability subtypes	Social media addiction increased significantly over one year (*p* = 0.02). Career networking via social media increased (*p* < 0.001). Male students showed a sharper rise in addiction than females (*p* < 0.05). Students with psychological disabilities increased career networking faster than those with physical disabilities (*p* < 0.05).
Ghaderi, 2024 [48]	To determine whether screen time, social networking use, and physical activity predict depression levels among Qazvin University students.	Social support and self-esteem	Among 146 undergraduates, greater daily screen time significantly correlated with higher depression scores (r = 0.35, *p* < 0.01) and independently predicted symptoms (β = 0.33, *p* = 0.001), explaining 13% of variance; social network use and physical activity showed no significant associations.
Helmy et al., 2024 [39]	To determine rates of alexithymia and its relationship with smartphone addiction and psychological distress among university students across Egypt, Oman, and Pakistan.	Alexithymia, smartphone addiction, and psychological distress	University students found 43% struggled to name emotions, 65% were addicted to smartphones, and 70% had high stress. Those with emotion-naming struggles were very likely to have phone addiction (*p* < 0.001) and distress (*p* < 0.001). Women had more emotion-naming issues than men (*p* < 0.001), and Oman had the highest phone addiction rate (*p* < 0.01).
Lerma & Cooper, 2024 [20]	To explore sociocultural, behavioral, and physical factors influencing excessive social media use, addiction, self-control failure, and motivation to reduce usage among Hispanic college students.	Self-control, self-esteem, and academic burnout	Social media addiction positively correlated with posting frequency in Spanish (*p* < 0.001), fear of missing out (*p* = 0.02), social media craving (*p* < 0.001), and home restrictions (*p* = 0.04). Motivation to reduce usage was higher among U.S. residents than Mexico (*p* = 0.05).
Li et al., 2024 [38]	To determine whether social withdrawal predicts problematic social media use in Chinese college students and to test alexithymia and negative body image as independent and chained mediators.	Social withdrawal, alexithymia, and negative body image	Among 2582 Chinese undergraduates, social withdrawal predicted greater problematic social media use (*p* < 0.001). Alexithymia and negative body image each partially mediated the link (*p* < 0.001) and combined in a significant chain (*p* < 0.001), explaining 42% of the indirect effect; the overall model fit was excellent
Oyinbo et al., 2024 [46]	To investigate the association between daily social media use and perceived stress among college students in the U.S.	Smartphone addiction and academic procrastination	Female students spending > 2 h/day on social media reported significantly higher stress levels compared to those spending ≤ 20 min/day (β = 4.74, 95% CI: 1.25–8.24, *p* < 0.05), highlighting prolonged social media use as an independent predictor of perceived stress.
Pi et al., 2024 [44]	To identify latent profiles of problematic mobile social media usage (PMSMU) among Chinese college students and assess factors such as FOMO, online feedback, and boredom.	Academic stress and usage duration	Three latent PMSMU profiles emerged: no-problem (26.44%), mild (56.66%), and severe (16.91%). Higher FOMO (OR = 2.91), boredom (OR = 8.72), and online positive feedback (OR = 1.42) significantly predicted severe PMSMU (*p* < 0.01 to *p* < 0.001). Females showed a higher risk (*p* < 0.001).
Sánchez-Fernández et al., 2024 [49]	To examine psychological factors associated with generalized problematic internet use (GPIU), problematic social media use (PSMU), and problematic online gaming (POG) among university students	Psychological distress, cognitive distortions, impulsiveness, and coping motives	High cognitive distortions (*p* < 0.001) and cognitive reappraisal (*p* < 0.01) were associated with GPIU, PSMU, and POG. Psychological distress, low conscientiousness, and motor impulsivity significantly predicted GPIU and PSMU (*p* < 0.05 to *p* < 0.001), but not POG. Neuroticism showed no significant association.
Shen et al., 2024 [29]	Examine the directionality between problematic mobile phone use (PMPU) symptoms and negative emotions in 5568 Chinese freshmen over one academic year using a cross-lagged panel network design	Academic burnout, social anxiety, escapism, and internet use motives	Baseline academic burnout predicted increased social-media and gaming use, plus all PMPU symptoms and negative emotions at follow-up (β = 0.01–0.04, *p* < 0.001). Bidirectional cycles linked escapism with social anxiety and inability to control craving (β ≈ 0.02–0.04, *p* < 0.01), with productivity loss emerging as the most central node.
Sirtoli et al., 2024 [30]	Estimate the association between time spent on social media (TSSM) and depressive symptoms in university students, and test whether tobacco, alcohol and illicit-drug use mediate that link	Tobacco and alcohol, illicit drug use	Among 3161 Brazilian students, longer daily social-media time predicted greater depressive symptoms (*p* < 0.001). Tobacco use (*p* = 0.02), alcohol risk (*p* < 0.001), and illicit-drug risk (*p* < 0.001) each partially mediated this association, together accounting for over two-thirds of the indirect effect after adjustment for age, sex, BMI, and physical activity.
Üzer et al., 2024 [36]	Examine how chronotype, psychological pain, problematic social-media use (PSMU), internet addiction, anxiety and depression interrelate with suicidality in Turkish university students, and test whether PSMU and psychological pain mediate the chronotype-suicidality link.	Evening chronotype and psychological pain	Among 571 students, higher psychological pain and PSMU independently predicted greater suicidality (*p* < 0.001). Evening chronotype was associated with higher suicidality (*p* = 0.009) and its effect was fully mediated by PSMU and psychological pain (*p* < 0.001); internet addiction, anxiety and depression showed no significant mediation.
Wang et al., 2024 [32]	To examine the longitudinal relationship between self-esteem and problematic social media use (PSMU) among Chinese college students.	Sleep quality, depressive, and anxiety symptoms	Self-esteem negatively predicted problematic social media use longitudinally (β = −0.151 to −0.132, *p* < 0.01 to *p* < 0.05). Initial self-esteem negatively predicted initial PSMU (β = −0.711, *p* < 0.01), and declining self-esteem predicted increasing PSMU (β = −0.708, *p* < 0.05).
Yan et al., 2024 [34]	To test whether the intensity of mobile social media use predicts depressive mood in college students and to determine if upward social comparison and cognitive overload act as mediators.	Perceived stress and academic stress	Among 568 Chinese students, greater social media use intensity was related to higher depressive mood (*p* < 0.001). Upward social comparison and cognitive overload each fully mediated this association (*p* < 0.01) and sequentially combined in a chain mediation (*p* < 0.001), nullifying the direct effect.
Yuan et al., 2024 [40]	To investigate longitudinal associations between negative life events and problematic social media use (PSMU) among Chinese college students, focusing on the mediating role of fear of missing out (FoMO) and moderation by positive parenting.	Social support and emotional intelligence	Negative life events increased PSMU directly and via higher FoMO (*p* < 0.001). Positive parenting significantly moderated the relationship between fear of missing social opportunities and PSMU (*p* = 0.019), indicating a protective effect against developing problematic social media behaviors.

Numerous studies reported statistically significant associations between social media use and mental health outcomes. For instance, Jameel et al. (2025) [31] found that social media addiction significantly predicted mental health issues (β = 0.315, *p* < 0.001) and insomnia (β = 0.537, *p* < 0.001). Sun and Tang (2025) [45] reported strong correlations between problematic smartphone use and depression (r = 0.451, *p* < 0.001), loneliness (r = 0.504, *p* < 0.001), and social media addiction (r = 0.614, *p* < 0.001). Yuan et al. (2024) [40] showed that negative life events predicted PSMU both directly and via FoMO (*p* < 0.001), with moderation by positive parenting (*p* = 0.019). These statistically significant results underscore the robustness of the psychological and contextual determinants identified in this review.

Across the 22 studies reviewed, 12 explicitly invoked at least one named theory, yielding 13 distinct frameworks overall. The most frequently cited were Uses & Gratifications Theory (n = 4) and Compensatory Internet Use Theory (n = 4), followed by Self-Determination Theory (n = 3) and Social Comparison Theory (n = 3). Less commonly referenced were Psychache Theory, Social Cognitive Theory, Ecological Systems Theory, Maslow’s Hierarchy of Needs, Attachment Theory, Social Learning Theory, Cognitive Overload Theory, and Dual-System Theory (each n = 1).

Alongside theoretical perspectives, the studies examined a range of determinants. Psychological factors such as depression, anxiety, FoMO, self-esteem, emotional intelligence, and loneliness were reported in 18 studies. Behavioral determinants including screen time, sleep quality, and academic stress were assessed in 15 studies, while demographic factors such as gender and age appeared in 10 studies. Contextual influences such as peer norms and sociocultural pressures were less frequently examined (6 studies). This distribution demonstrates that psychological and behavioral determinants dominate current research, while demographic and contextual factors remain underexplored, and theoretical frameworks are unevenly applied.

Cross-study synthesis revealed consistent links between excessive social media use and poor mental health outcomes, yet contradictory evidence also emerged. For instance, while some studies identified peer support as protective, this was highlighted by Fruehwirth et al. [33]. others suggested that peer-driven norms reinforced compulsive use, as mentioned by Sun and Tang [45]. Similarly, although academic stress was consistently associated with problematic use, its mediating role varied across contexts. These contradictions point to a lack of standardized measurement and theory-driven analysis in the current literature.

## 4. Discussion

The findings of the review reveal a significant interrelation between problematic social media use (PSMU) among university students and various psychological outcomes such as anxiety, depression, and emotional dysregulation. Consistently, studies indicate that overuse of social media platforms is linked to increased mental health issues, suggesting that students frequently engage in social comparison and experience feelings of missing out (FoMO), which exacerbate emotional distress [50].

Research has further shown that psychological constructs, including emotional intelligence and self-esteem, play critical roles in mediating these relationships. For instance, low self-esteem is recognized as a predictor of PSMU, corroborating findings from previous literature on this subject [48]. Additionally, factors such as social media fatigue and excessive screen time are noted to correlate strongly with poor sleep quality, thereby compounding emotional difficulties [40].

Furthermore, the contextual implications of academic stress highlight that environmental pressures contribute significantly to PSMU, reinforcing previous assertions about the negative impact of stressors on digital engagement [51]. This emphasizes the need for integrated approaches in addressing PSMU within educational environments, particularly in fostering healthier digital habits among students [52]. Thus, these findings call for enhanced awareness and intervention strategies to mitigate the risks associated with excessive social media exposure, particularly in academic settings [53].

Compared to earlier systematic reviews, such as Keles et al. [25] this scoping review, which primarily examined associations between social media use and mental health outcomes, this scoping review offers a distinct contribution by focusing specifically on the behavioral, demographic, and psychosocial determinants of social media use among college students. Unlike previous reviews, our study highlights the absence of theoretical framing in many existing studies and identifies key constructs that could inform future intervention design. Notably, across the 22 included studies, 12 explicitly employed at least one theoretical framework, though application was uneven. Uses & Gratifications Theory and Compensatory Internet Use Theory were the most frequently applied (n = 4 each), followed by Self-Determination Theory and Social Comparison Theory (n = 3 each). A wide array of other theories appeared only once, underscoring the fragmented and inconsistent use of theory in this field. This highlights the need for greater integration of frameworks to guide future research and strengthen explanatory depth. Additionally, this review includes recent studies from 2024–2025, capturing evolving trends in digital engagement within the higher education context.

### 4.1. Uses of Social Media for Changing Health Behaviors

Social media platforms exert a significant influence on health behavior change among university students, serving both as a means of promoting awareness and facilitating community support. During periods of social restrictions, such as those experienced during the COVID-19 pandemic, online communication became crucial for youth engagement and well-being [54]. This environment fostered increased utilization of digital platforms for health advocacy and information dissemination, which can effectively alter health behaviors.

Research indicates that various digital health interventions have successfully leveraged social media to improve aspects of physical and mental health [55]. For instance, platforms used for health promotion provide resources that encourage physical activity, enhance emotional resilience, and promote healthy coping mechanisms [32]. The convenience of accessing health information via familiar technologies serves to lower barriers to the self-management of health conditions [56].

Additionally, the role of social media in facilitating supportive networks is crucial. Engaging with peers on health-related topics through digital platforms creates a sense of community, which can be vital for maintaining motivation and accountability in health behavior change efforts. However, to maximize the potential of social media in this domain, it is essential to cultivate digital literacy and critical thinking about online health information among students to encourage informed health choices [57].

### 4.2. Strengths of the Studies

These 22 studies demonstrate methodological rigor through large, multi-site samples (n = 389–5568) across diverse cultural settings. Most employed validated addiction and psychological scales, with two studies refining instruments [45] and three using longitudinal designs to strengthen causal inference. Advanced analytics, such as network modeling [29], enhanced understanding of symptom interplay. The inclusion of both cross-sectional and longitudinal surveys, psychometric validation, and multi-country comparisons enhances generalizability and supports nuanced insights into social-media determinants and mental-health outcomes.

### 4.3. Limitations of the Studies

Notwithstanding their contributions, these studies are constrained by predominantly cross-sectional designs (20/22), which preclude definitive causal inferences. Convenience sampling from a single university limits representativeness and may introduce selection bias. Heterogeneity in measurement—varying scales, cut-offs, and time-frames—hampered direct comparisons and meta-analytic synthesis. Self-reported usage and symptom data are susceptible to recall and social-desirability biases. Moreover, scant theoretical grounding in many papers undermines conceptual coherence, and the under-representation of experimental or ecological-momentary assessment methods limits ecological validity (Breton & de Leeuw, 2010) [58].

### 4.4. Strengths of the Review

This scoping review adhered to PRISMA-ScR guidelines and implemented a transparent, reproducible protocol across three major databases (MEDLINE, CINAHL, ERIC), ensuring comprehensive capture of 2024–2025 literature. Dual independent screening and standardized data charting minimized selection bias and enhanced methodological rigor. Focusing on recent studies provided up-to-date insights into evolving platform dynamics. Inclusion of both cross-sectional and longitudinal designs, and cross-cultural comparisons supported broad generalizability. Finally, synthesizing cognitive-effective and contextual determinants offers a nuanced framework for future theory-driven research.

### 4.5. Limitations of the Review

This scoping review has several important limitations. First, we restricted our search to three bibliographic databases (MEDLINE, CINAHL, and ERIC) and peer-reviewed articles published in English, potentially omitting relevant studies in other languages, gray literature, or non-indexed sources. Second, the review covered a relatively short publication window (January 2024–May 2025), which may limit the assessment of longer-term trends in social media use among college students. Third, no formal appraisal of methodological quality was performed using a standardized risk-of-bias assessment tool (e.g., ROBIS or JBI Checklist). As such, the potential for methodological flaws in the included studies could not be systematically evaluated. This limits the ability to fully assess the strength and reliability of the findings and may introduce bias in the synthesis. Most included studies also employed cross-sectional designs with self-reported measures, further constraining causal interpretation and raising the possibility of response and recall bias. Due to the heterogeneity in study designs, populations, and outcome measures, and the nature of a scoping review, a meta-analysis or visual synthesis (e.g., forest or funnel plots) was not conducted.

### 4.6. Implications for Practice

The findings of this scoping review underscore the necessity for integrated interventions aimed at addressing the determinants of problematic social media use (PSMU) among college students. Given the documented relationship between excessive social media engagement and negative psychological outcomes such as anxiety, depression, and emotional dysregulation [59,60]. Universities should prioritize mental health resources tailored to assist students in navigating digital environments. Mental health services should incorporate strategies that emphasize media literacy, helping students critically assess their online habits and recognize the impacts of digital engagement on their mental well-being [53,54].

Moreover, the promotion of healthy digital behaviors can benefit from empirical evidence highlighting the role of peer support and community engagement [60]. Initiating student-led wellness campaigns could foster a culture of support, encourage positive social media interactions, and promote resilience against adverse outcomes associated with social media overuse. For instance, interventions that focus on education around issues like fear of missing out (FoMO) and self-esteem can equip students with the skills necessary to engage with social media more healthily [59].

Additionally, fostering partnerships with technology providers to develop screen-time monitoring applications may play a crucial role in encouraging self-regulation among students. The implementation of these applications can provide students with insights into their usage patterns, ultimately fostering a more balanced approach to media consumption [61]. In summary, effective strategies should be multidimensional, encompassing education, peer support, and practical tools, to promote healthy digital engagement among college students.

The findings underscore a recurring pattern: students often recognize negative emotional outcomes but continue excessive use, suggesting dissonance between awareness and behavior. This contradiction highlights the need for interventions that move beyond education and emphasize emotional regulation, peer norms, and behavioral control. Policies must also consider platform-level changes and institutional support systems to reduce digital overload and promote healthy digital environments.

For educators, integrating structured digital wellness modules into orientation or first-year seminars could help students recognize and regulate their online behaviors. For policymakers, mandating digital literacy curricula at the institutional level and supporting national campaigns may reduce risky engagement. For researchers, adopting longitudinal designs grounded in models such as MTM or SDT will improve causal inference and ensure findings are translatable into interventions. Together, these targeted recommendations provide concrete steps for addressing problematic social media use across multiple levels.

### 4.7. Recommendations for Future Research

Very few studies on determinants utilized theoretical frameworks. Hence, there is a need to utilize contemporary frameworks such as the multi-theory model (MTM) of health behavior change to understand the correlates of social media use among college students [62,63]. In addition to MTM, several classical theories may also help explain the observed behavioral patterns. The Health Belief Model (HBM), for example, can offer insights into perceived susceptibility and benefits associated with reduced social media use. The Theory of Planned Behavior (TPB) may explain students’ intentions and perceived behavioral control over social media habits, while Self-Determination Theory (SDT) can contextualize how autonomy, competence, and relatedness need drive digital engagement. Integrating these models in future research can strengthen the explanatory power of studies and inform the design of effective, theory-driven interventions. To effectively address smartphone addiction and its impact on college students, institutions should implement comprehensive wellness programs that focus on enhancing media literacy and promoting healthy digital habits. Strategies should include workshops about the risks associated with excessive social media use, such as anxiety and depression, particularly amidst disruptive events like the COVID-19 pandemic [64]. Additionally, fostering environments that encourage peer support and community engagement can help mitigate feelings of isolation and enhance students’ psychological well-being [65]. Furthermore, developing screening tools to help identify students at risk for addiction or mental health issues can be instrumental in promoting timely interventions. In light of the complex relationship between social media use and mental health, institutional policies should also advocate for monitored and balanced usage to improve overall student engagement and academic performance [66].

## 5. Conclusions

This scoping review synthesized evidence from 22 studies on college students’ social media use and identified key behavioral determinants (e.g., screen time, FoMO), demographic factors (e.g., gender, disability status), and psychosocial correlates (e.g., self-esteem, emotional intelligence) linked to mental health outcomes such as anxiety, depression, and sleep disturbances. Among these studies, 12 explicitly employed at least one theoretical framework, most often Uses & Gratifications Theory and Compensatory Internet Use Theory (n = 4 each), followed by Self-Determination Theory and Social Comparison Theory (n = 3 each). A wider range of additional theories appeared only once, underscoring the fragmented and inconsistent use of theory in this field. These findings indicate that while determinants are well documented, theoretical integration remains limited. Heterogeneity in methods and reliance on cross-sectional designs constrain causal inference. Future research should adopt theory-driven frameworks, longitudinal and experimental designs, and standardized measures to strengthen explanatory depth and guide evidence-based interventions. Collectively, these insights provide actionable recommendations for educators, policymakers, and researchers seeking to foster healthier digital engagement among students.

## Figures and Tables

**Table 1:** Criteria sheet: key research terms.

Term	Associated Terms	Exclusions
Social media	“Social media”, “social networking sites”, “online social networks”	Studies not focused on social media platforms
College Students	“College students”, “university students”	Non-college youth, adults outside the college/university context
Determinants/Factors/Influences	“determinants”, “factors”, “influences”	Studies not addressing specific influences on social media use
Use/Usage/Engagement/Behavior	“use”, “usage”, “engagement”, “behavior”	Studies that do not focus on social media usage or engagement

## Data Availability

No new data were created or analyzed in this study. Data sharing does not apply to this article.

## Abbreviations

The following abbreviations are used in this manuscript:

TLA	Three-letter acronyms
FoMO	Fear of missing out
PSMU	Problematic social media use

## References

[1] Gottfried M.A. (2023). Michelle Faverio and Jeffrey Teens, Social Media and Technology 2023.

[2] Duong C.T., Lebret R., Aberer K. (2017). Multimodal Classification for Analysing Social Media. arXiv.

[3] Kaplan A.M., Haenlein M. (2010). Users of the World, Unite! The Challenges and Opportunities of Social Media. Bus. Horiz..

[4] Chugh R., Ruhi U. (2018). Social Media in Higher Education: A Literature Review of Facebook. Educ. Inf. Technol..

[5] Kolhar M., Kazi R.N.A., Alameen A. (2021). Effect of Social Media Use on Learning, Social Interactions, and Sleep Duration among University Students. Saudi J. Biol. Sci..

[6] Boyd D.M., Ellison N.B. (2007). Social Network Sites: Definition, History, and Scholarship. J. Comput.-Mediat. Commun..

[7] Maryville U. (2020). The Evolution of Social Media: How Did It Begin, and Where Could It Go Next.

[8] Reid Chassiakos Y.L., Radesky J., Christakis D., Moreno M.A., Cross C., Hill D., Ameenuddin N., Hutchinson J., Levine A., Council on Communications and Media (2016). Children and Adolescents and Digital Media. Pediatrics.

[9] Pempek T.A., Yermolayeva Y.A., Calvert S.L. (2009). College Students’ Social Networking Experiences on Facebook. J. Appl. Dev. Psychol..

[10] Abi-Jaoude E., Naylor K.T., Pignatiello A. (2020). Smartphones, Social Media Use and Youth Mental Health. Can. Med Assoc. J..

[11] Koç T., Turan A.H. (2021). The Relationships Among Social Media Intensity, Smartphone Addiction, and Subjective Wellbeing of Turkish College Students. Appl. Res. Qual. Life.

[12] Barry C.T., Moran-Miller K., Levy H.F., Gray T. (2024). Social Media Engagement, Perceptions of Social Media Costs and Benefits, and Well-Being in College Student-Athletes. J. Am. Coll. Health.

[13] Gómez-Galán J., Martínez-López J.Á., Lázaro-Pérez C., Sarasola Sánchez-Serrano J.L. (2020). Social Networks Consumption and Addiction in College Students during the COVID-19 Pandemic: Educational Approach to Responsible Use. Sustainability.

[14] Hruska J., Maresova P. (2020). Use of Social Media Platforms among Adults in the United States—Behavior on Social Media. Societies.

[15] Abbas J., Aman J., Nurunnabi M., Bano S. (2019). The Impact of Social Media on Learning Behavior for Sustainable Education: Evidence of Students from Selected Universities in Pakistan. Sustainability.

[16] Zhuang J., Mou Q., Zheng T., Gao F., Zhong Y., Lu Q., Gao Y., Zhao M. (2023). A Serial Mediation Model of Social Media Addiction and College Students’ Academic Engagement: The Role of Sleep Quality and Fatigue. BMC Psychiatry.

[17] Clark D.L., Raphael J.L., McGuire A.L. (2018). HEADS4: Social Media Screening in Adolescent Primary Care. Pediatrics.

[18] Albikawi Z.F. (2023). Anxiety, Depression, Self-Esteem, Internet Addiction and Predictors of Cyberbullying and Cybervictimization among Female Nursing University Students: A Cross Sectional Study. Int. J. Environ. Res. Public Health.

[19] Aldhawyan A.F., Alfaraj A.A., Elyahia S.A., Alshehri S.Z., Alghamdi A.A. (2020). Determinants of Subjective Poor Sleep Quality in Social Media Users Among Freshman College Students. Nat. Sci. Sleep.

[20] Lerma M., Marquez C., Sandoval K., Cooper T.V. (2021). Psychosocial Correlates of Excessive Social Media Use in a Hispanic College Sample. Cyberpsychol. Behav. Soc. Netw..

[21] Bekalu M.A., Sato T., Viswanath K. (2023). Conceptualizing and Measuring Social Media Use in Health and Well-Being Studies: Systematic Review. J. Med. Internet Res..

[22] Tuck A.B., Thompson R.J. (2024). The Social Media Use Scale: Development and Validation. Assessment.

[23] Shimoga S.V., Erlyana E., Rebello V. (2019). Associations of Social Media Use With Physical Activity and Sleep Adequacy Among Adolescents: Cross-Sectional Survey. J. Med. Internet Res..

[24] Duggan M. (2015). Mobile Messaging and Social Media 2015.

[25] Keles B., McCrae N., Grealish A. (2020). A Systematic Review: The Influence of Social Media on Depression, Anxiety and Psychological Distress in Adolescents. Int. J. Adolesc. Youth.

[26] Moorhead S.A., Hazlett D.E., Harrison L., Carroll J.K., Irwin A., Hoving C. (2013). A New Dimension of Health Care: Systematic Review of the Uses, Benefits, and Limitations of Social Media for Health Communication. J. Med. Internet Res..

[27] Page M.J., McKenzie J.E., Bossuyt P.M., Boutron I., Hoffmann T.C., Mulrow C.D., Shamseer L., Tetzlaff J.M., Akl E.A., Brennan S.E. (2021). The PRISMA 2020 Statement: An Updated Guideline for Reporting Systematic Reviews. BMJ.

[28] Purba A.K., Thomson R.M., Henery P.M., Pearce A., Henderson M., Katikireddi S.V. (2023). Social Media Use and Health Risk Behaviours in Young People: Systematic Review and Meta-Analysis. BMJ.

[29] Shen G., Huang G., Wang M., Jian W., Pan H., Dai Z., Wu A.M.S., Chen L. (2024). The Longitudinal Relationships between Problematic Mobile Phone Use Symptoms and Negative Emotions: A Cross-Lagged Panel Network Analysis. Compr. Psychiatry.

[30] Sirtoli R., Fernández-Rodríguez R., Balboa-Castillo T., Rodrigues R., Garrido-Miguel M., Eumann Mesas A., Morales G., Molino Guidoni C. (2024). Time Spent on Social Media and Depressive Symptoms in University Students: The Mediating Role of Psychoactive Substances. Am. J. Addict..

[31] Jameel A., Guo W., Hussain A., Kanwel S., Sahito N. (2025). Exploring the Mediating Role of Insomnia on the Nexus between Social Media Addiction and Mental Health among University Students. Sci. Rep..

[32] Wang H. (2024). Relationship Between Self-Esteem and Problematic Social Media Use Amongst Chinese College Students: A Longitudinal Study. Psychol. Res. Behav. Manag..

[33] Fruehwirth J.C., Weng A.X., Perreira K.M. (2024). The Effect of Social Media Use on Mental Health of College Students during the Pandemic. Health Econ..

[34] Yan N., Long Y., Yuan H., Zhou X., Xie B., Wang Y. (2024). The Impact of Mobile Social Media Use on Depressive Mood Among College Students: A Chain Mediating Effect of Upward Social Comparison and Cognitive Overload. Psychol. Res. Behav. Manag..

[35] Thomas R., George M.A. (2025). Fear of Missing out (FOMO), Emotional Distress, and Problematic Social Media Use among University Student. Indian. J. Psychiatr. Soc. Work..

[36] Üzer A., Uran C., Yılmaz E., Şahin Ş.N., Ersin M.K., Yılmaz R.H., Çıkla A. (2024). The Relationship between Chronotype, Psychological Pain, Problematic Social Media Use, and Suicidality among University Students in Turkey. Chronobiol. Int..

[37] Serdar Eymirli P., Mustuloğlu Ş., Köksal E., Turgut M.D., Uzamiş Tekçiçek M. (2024). Dental Students’ Healthy Lifestyle Behaviors, Physical Activity Levels and Social Media Use: Cross Sectional Study. Discov. Public Health.

[38] Li S., Chen X., Liu L., Sun C. (2024). The Relationship between Social Withdrawal and Problematic Social Media Use in Chinese College Students: A Chain Mediation of Alexithymia and Negative Body Image. BMC Psychol..

[39] Helmy M., Ebrahim A.H., Faqeeh A., Engel E., Ashraf F., Isaac B.A. (2024). Relationship Between Alexithymia, Smartphone Addiction, and Psychological Distress Among University Students: A Multi-Country Study. Oman Med. J..

[40] Yuan X., Dou K., Li Y. (2024). The Longitudinal Association Between Negative Life Events and Problematic Social Media Use Among Chinese College Students: The Mediating Role of FoMO and the Moderating Role of Positive Parenting. Stress. Health.

[41] Rahman T., Kim Y.S., Noh M., Lee C.K. (2021). A Study on the Determinants of Social Media Based Learning in Higher Education. Educ. Technol. Res. Dev..

[42] Bawazeer N.M., Almalki S., Alanazi R., Alamri R., Alanzi R., Alhanaya R., Alhashem A., Aldahash R. (2025). Examining the Association between Social Media Use and Dietary Habits among College Students in Riyadh, Saudi Arabia. J. Community Health.

[43] Gao N., Eissenstat S.J., DeMasi M. (2024). A One-Year Follow-up Study of Changes in Social Media Addiction and Career Networking among College Students with Disabilities. J. Am. Coll. Health.

[44] Pi L., Wang Y., Zou L., Mo X., Guo L. (2024). An Analysis of the Latent Class and Influencing Factors of Problematic Mobile Social Media Usage Among Chinese College Students. Psychol. Res. Behav. Manag..

[45] Sun H., Tang K. (2025). Psychometric Evaluation and Measurement Invariance of the Problematic Smartphone Use Scale among College Students: A National Survey of 130 145 Participants. Addiction.

[46] Oyinbo A.G., Heavner K., Mangano K.M., Morse B., El Ghaziri M., Thind H. (2024). Prolonged Social Media Use and Its Association with Perceived Stress in Female College Students. Am. J. Health Educ..

[47] Karaduman C. (2025). The Relationship Between Social Media Addiction and Mental Fatigue Levels in Faculty of Health Sciences Students: A Descriptive and Relational Study. J. Educ. Res. Nurs..

[48] Ghaderi D. (2024). The Relationship Between Screen Time, Social Network Usage, and Physical Activity with Depression in Students at Qazvin University. Asian J. Sports Med..

[49] Sánchez-Fernández M., Borda-Mas M., Horvath Z., Demetrovics Z. (2024). Similarities and Differences in the Psychological Factors Associated with Generalised Problematic Internet Use, Problematic Social Media Use, and Problematic Online Gaming. Compr. Psychiatry.

[50] Qin C., Li Y., Wang T., Zhao J., Tong L., Yang J., Liu Y. (2024). Too Much Social Media? Unveiling the Effects of Determinants in Social Media Fatigue. Front. Psychol..

[51] Dogra N., Sharma S. (2024). Smartphone Use and Its Addiction among Jammu Adolescents. Indian. J. Community Health.

[52] Tu W., Nie Y., Liu Q. (2023). Does the Effect of Stress on Smartphone Addiction Vary Depending on the Gender and Type of Addiction?. Behav. Sci..

[53] Alhafi M., Matrood R., Alamoudi M., Alshaalan Y., Alassafi M., Omair A., Harthi A., Layqah L., Althobaiti M., Shamou J. (2024). The Association of Smartphone Usage with Sleep Disturbances among Medical Students. Avicenna J. Med..

[54] Lowthian E., Fee G., Wakeham C., Clegg Z., Crick T., Anthony R. (2024). Identifying Protective and Risk Behavior Patterns of Online Communication in Young People. J. Adolesc..

[55] Hegeman P., Vader D., Kamke K., El-Toukhy S. (2024). Patterns of Digital Health Access and Use among US Adults: A Latent Class Analysis. BMC Digit. Health.

[56] Kirsch E.P., Kunte S.A., Wu K.A., Kaplan S., Hwang E.S., Plichta J.K., Lad S.P. (2024). Digital Health Platforms for Breast Cancer Care: A Scoping Review. J. Clin. Med..

[57] Doelvia A., Hien V.T.T., Rathee S. (2023). Assessment: The Effectiveness of Video Media Through the Tiktok Application on Teenagers’ Knowledge About Clean and Healthy Living Behavior at Junior High School Level. J. Eval. Educ..

[58] Breton E., De Leeuw E. (2011). Theories of the Policy Process in Health Promotion Research: A Review. Health Promot. Int..

[59] Zhu C., Li S., Zhang L. (2025). The Impact of Smartphone Addiction on Mental Health and Its Relationship with Life Satisfaction in the Post-COVID-19 Era. Front. Psychiatry.

[60] Sun C. (2024). Analysis Of the Impact of New Media on College Students’ Mental Health. Educ. Humanit. Soc. Sci..

[61] Hatmanti N.M., Septianingrum Y., Fitriasari A., Martining Wardani E., Setiyowati E. (2024). Smartphone Addiction Screening Application Development Based on Android: A Preeliminary Study. E3S Web Conf..

[62] Sharma M., Awan A., Kapukotuwa S., Kanekar A., Liamputtong P. (2024). A Fourth-Generation Multi-Theory Model (MTM) of Health Behavior Change: Genesis, Evidence, and Potential Applications. Handbook of Concepts in Health, Health Behavior and Environmental Health.

[63] Kapukotuwa S., Nerida T., Batra K., Sharma M. (2024). Utilization of the Multi-Theory Model (MTM) of Health Behavior Change to Explain Health Behaviors: A Systematic Review. Health Promot. Perspect..

[64] Bailey E., Boland A., Bell I., Nicholas J., La Sala L., Robinson J. (2022). The Mental Health and Social Media Use of Young Australians during the COVID-19 Pandemic. Int. J. Environ. Res. Public Health.

[65] Serra G., Lo Scalzo L., Giuffrè M., Ferrara P., Corsello G. (2021). Smartphone Use and Addiction during the Coronavirus Disease 2019 (COVID-19) Pandemic: Cohort Study on 184 Italian Children and Adolescents. Ital. J. Pediatr..

[66] Lee D.S., Way B.M. (2021). Social Media Use and Systemic Inflammation: The Moderating Role of Self-Esteem. Brain Behav. Immun.-Health.

